# Unilateral fatiguing exercise and its effect on ipsilateral and contralateral resting mechanomyographic mean frequency between aerobic populations

**DOI:** 10.14814/phy2.13151

**Published:** 2017-02-27

**Authors:** Nathan P. Wages, Travis W. Beck, Xin Ye, Joshua C. Carr

**Affiliations:** ^1^ Department of Health and Exercise ScienceUniversity of OklahomaNormanOklahoma; ^2^ Department of HealthExercise Science and Recreation ManagementUniversity of MississippiUniversityMississippi

**Keywords:** Aerobic fatiguing exercise, Postural positions, Unilateral exercise

## Abstract

The purpose of this investigation was to establish a better understanding of contralateral training and its effects between homologous muscles following unilateral fatiguing aerobic exercise during variable resting postural positions, and to determine if any observable disparities could be attributed to the differences between the training ages of the participants. Furthermore, we hypothesized that we would observe a contralateral cross‐over effect for both groups, with the novice trained group having the higher mechanomyographic mean frequency values in both limbs, across all resting postural positions. Twenty healthy male subjects exercised on an upright cycle ergometer, using only their dominate limb, for 30 min at 60% of their VO_2_ peak. Resting electromyographic and mechanomyographic signals were measured prior to and following fatiguing aerobic exercise. We found that there were resting mechanomyographic mean frequency differences of approximately 1.9 ± 0.8% and 0.9 ± 0.7%; 9.1 ± 0.3% and 10.2 ± 3.7%; 2 ± 1.8% and 3 ± 1.4%; and 0.9 ± 0.6% and 0.2 ± 1.3% between the novice and advanced trained groups (for the upright sitting position with legs extended 180°; upright sitting position with legs bent 90°; lying supine position with legs extended 180°; and lying supine with legs bent 90°, respectively), from the dominant and nondominant limbs, respectively. We have concluded that despite the relative matching of exercise intensity between groups, acute responses to contralateral training become less accentuated as one progresses in training age. Additionally, our results lend support to the notion that there are multiple, overlapping neural and mechanical mechanisms concurrently contributing to the contralateral cross‐over effects observed across the postexercise resting time course.

## Introduction

The term “cross‐over effect” has been defined as the acute response to a potential “cross‐educational effect”, which has been linked with adaptations due to repeated bouts of exercise over an extended period of training (Weir et al. 1994; Carroll et al. 2006; Toca‐Herrera et al. 2008; Wages et al. 2015, 2016a). Thus, as an individual progressively exercises across an extended period of time, the neural adaptations that were once prominent start to steadily decline, while the subsequent hypertrophic adaptations begin to become more eminent (Lee and Carroll 2007; Hortobágyi et al. 2011; Farthing and Zehr 2014). It is at this same time that the interlateral effectual term transitions from “cross‐over” to “cross‐education” (e.g., potentially following, approximately 4–8 weeks of repeated bout training [Shaver 1975; Munn et al. 2004]). Furthermore, regardless of both terms explaining the same relative interlateral event across training time, they are both related to the same unilateral exercise modality, “contralateral training”. Contralateral training has been defined as the act of exercising (a) particular muscle(s) from one appendage to enhance acute responses or chronic adaptations in the homologous muscle(s) from the opposing limb (Lee et al. 2009; Hortobágyi et al. 2011; Doix et al. 2013; Farthing and Zehr 2014). Interestingly, the act of training asymmetrically is not a relatively new exercise modality, due to its investigation being first published in the early 1890s (Scripture et al. 1894). However, despite its continuous investigation, there is still conflicting and inconclusive evidence regarding which neurological mechanism(s) (i.e., cortical, subcortical/supraspinal, spinal, or segmental [Hortobágyi et al. 1997; Lee and Carroll 2007; Adamson et al. 2008; Starbuck and Eston 2012]) primarily control this form of training modality. Recently, few researchers (Carroll et al. 2006; Hortobágyi et al. 2011) have begun to hypothesize that there is a collaborative effort amongst multiple neurological (in conjunction with mechanical) mechanisms facilitating these responses and adaptations across acute and chronic training periods, respectively. Yet, despite their compelling hypotheses, it appears that these authors could not determine if this collaborative effort, during either the acute response or chronic adaptation, changes between different neurological mechanisms, or if the same neurological mechanism(s) remains completely active throughout the entire training process. Additionally, these authors did note that whether assessing the acute responses or chronic adaptations, the respective limb that is used to perform the unilateral training is of relative importance.

In the recent past, numerous investigators (Sainburg and Wang 2002; Criscimagna‐Hemminger et al. 2003; Wang and Sainburg 2003, 2004; Hortobágyi 2005; Farthing et al. 2007; Hinder et al. 2013) have evaluated the sequencing order between homologous limbs during unilateral training. What these authors found was that acute limb responses or chronic adaptations across time are explicitly interlimb dependent. Hence, larger increases in positive muscular responses or adaptations are observed when the dominant (DOM) limb is primarily trained, instead of when the nondominant (N‐DOM) limb is trained (Davies et al. 1988; Housh et al. 1992; Farthing et al. 2005; Farthing 2009; Farthing and Zehr 2014). Thus, these previous researchers have suggested that when the N‐DOM limb is trained (instead of the DOM limb) there is little‐to‐no significant levels of improvement (except when learning a new muscular action for the very first time). Therefore, when reporting limb differences, it would be inappropriate to only list exercised verses non‐exercised limbs (EXL and N‐EXL, respectively) without giving reference to which limb (either DOM or N‐DOM) performed the specified action during exercise.

In addition, researchers need to also be cognizant to the particular joint angle in which an appendage is placed following the conclusion of exercise. Depending on the specified joint angle, the associated resting musculature could be in the lengthened or shortened state, which would have an effect on its recovery rate, as well as on the associated mechanomyographic (MMG) signals being recorded. For example, “traditional theory” would suggest that if a muscle were to be lengthened, the result should be a high level of muscle stiffness restricting the ability of those muscle fibers to oscillate, thereby decreasing the MMG amplitude (AMP) and increasing the associated MMG mean frequency (MNF). Reciprocally, if that same muscle were to be shortened, the result should be that the passive stiffness would decrease, which would allow the muscle fibers to oscillate more freely, thus causing an increase in MMG AMP and a decrease in MMG MNF. However, previous researchers (Jaskólska et al. 2003; Wages et al. 2016a,b) have shown that this line of reasoning may not always be accurate when assessing resting musculature at different joint angles due, in part, to the type of fatigue experienced by those previously trained muscles (i.e., low‐ vs. high‐intensity bouts, short‐ vs. long‐term durations, etc.). Specifically, it has been well established that muscle fatigue causes confounding results to the frequency component of the MMG signal (Madeleine et al. 2002; Blangsted et al. 2005; Beck et al. 2007). More specifically, recent investigators (Weir et al. 2000; Itoh et al. 2004; Wages et al. 2016b) have observed that as a muscle becomes increasingly fatigued during exercise, the associated MMG MNF values decrease as a result. Yet, if that same muscle were to be less fatigued during exercise (or potentially recovering from fatiguing exercise), we would expect to observe a greater increase in the MMG MNF values.

Furthermore, it is also important to clarify that the act of examining a muscle's electrical and/or mechanical activity during exercise can be relatively challenging, but to examine that same muscle's activity following the cessation of exercise is quite demanding (due to the basal level of muscular activity that can be recorded). Thus, evaluating muscular activity during a state of rest is essential for interpreting certain acute responses or chronic adaptations associated with a particular muscle, or a group of muscles (i.e., fiber type, architectural arrangement, training, etc. [Beck et al. 2007; Wages et al. 2015, 2016a). For example, what would be the effectual magnitude of a contralateral cross‐over effect between advanced versus novice trained individuals for an appendicular limb? Well, “conventional wisdom” would suggest that the novice group would be more adept to greater cross‐over effects postexercise (due to the lower degree of “stimulus” needed for the specified response to occur). Therefore, as an individual transitions from novice to advance (based upon training age), their muscles should increase their functional capacity to conform to the “stressor” needed to create a response or adaptation. Hence, once an acute response or chronic adaptation has occurred, that particular muscle which was previously trained will now require an increased “stressor or stimulus” (using the overload principle [Hellebrandt and Houtz 1956; Higbie et al. 1996]) to produce a new desired response or adaptation (due to the acquisition of a higher level of fitness). However, if there is a relative increase in the “stressor or stimulus” needed for a particular response or adaptation to occur within each training group, shouldn't that response or adaptation be similar between both training groups? Unfortunately, due to a lack of literature regarding the elucidation of resting cross‐over effects between different training populations, there are presently no definitive answers for this particular question.

To assist with evaluating potential acute responses or chronic adaptations between training groups, previous researchers (Stokes and Dalton 1991; Ebersole et al. 2002; Beck et al. 2005; Chen et al. 2014) have suggested simultaneously recording the electrical and mechanical activity of a particular muscle in order to obtain a more complete description of the neuromuscular mechanism(s) that is (are) present during, or following, exercise. Specifically, the combinational recording of MMG and surface electromyography (sEMG) allows for the examination of the mechanical and electrical aspects of muscle function to monitor the dissociation between their respective events (Beck et al. 2012). More specifically, sEMG is a noninvasive tool to aid with examining a motor unit's electrical signal, while MMG is a noninvasive tool to aid with examining a motor unit's mechanical signal (e.g., deformations in microscopic vibrations, or lateral oscillations, from active motor units), from a consciously (or subconsciously) active (or resting) muscle (Jaskólska et al. 2003; Orizio et al. 2003; McKay et al. 2004; Wages et al. 2015). Furthermore, the MMG signal is considered by many researchers (Gordon and Holbourn 1948; Orizio et al. 1992, 2003; Beck 2010) to be the intrinsic mechanical counterpart to the motor unit's electrical signal. Additionally, unlike sEMG signals, the reliability of the MMG signal is not affected by the quality of sensor–skin interface (e.g., skin impedance, sweat accumulation, etc.), or location of an innervation zone within a muscle (Xie et al. 2009; Malek and Coburn 2011). However, despite the MMG sensor appearing to possess a greater sensitivity to that observed with the sEMG sensor (McKay et al. 2004, 2013), there are still a few factors that can potentially affect the MMG signal (i.e., musculature fatigue, muscle temperature; muscle stiffness; muscle and tendon lengths, muscle/adipose mass; intramuscular pressure; or viscosity of the intracellular and/or extracellular fluid mediums [Marchetti et al. 1992; Orizio and Veicsteinas 1992; Orizio 1993; Stokes 1993; Orizio et al. 2003]). Nevertheless, findings from previous investigations (Orizio et al. 1990, 2003; Orizio 1993; Akataki et al. 2001; Beck et al. 2007; Kawczynski et al. 2008) have also shown that the frequency component of the MMG signal provides valid, qualitative information regarding the global rate coding paradigm (e.g., global motor unit firing rate properties) from a multitude of active motor units (instead of only one or a few motor units, as previously believed).

Therefore, simultaneous measurements of the MMG and sEMG signals will be useful for potentially identifying the mechanical versus neural contributions to unilateral fatiguing exercise, respectively. Furthermore, an improved understanding of these mechanisms could eventually lead to the development of training strategies, or programs, that improve the functional performance, or the reacquisition of motor function, for the deficient, homologous limb. Thus, our primary purpose for this investigation was to establish a better understanding of contralateral training and its effects between ipsilateral and contralateral homologous muscles following unilateral fatiguing aerobic exercise during variable resting postural positions (RPPs) that incorporated modifications of hip and knee joint angles. Our secondary purpose was to determine if any observable disparities could be attributed to the differences between the fitness levels (based upon training age) of the participants. To aid with assessing the MMG and sEMG responses to acute exercise, we explained each group's ipsilateral and contralateral limb results related to their respective DOM, EXL, compared to their N‐DOM, N‐EXL. The reason was to use the N‐DOM limb as a matched reference control. Additionally, we had hypothesized (*a priori*) that we would observe a cross‐over effect for both groups, with the novice group having the higher MMG MNF values in both limbs, across all RPPs. Also, it is important to mention that this investigation is a follow‐up to our previous work (Wages et al. 2016a), which incorporated a relatively, untrained/sedentary population.

## Materials and Methods

### Ethical approval

This study conformed to the standards set by the Declaration of Helsinki, was approved by the University Institutional Review Board for Human Subjects, and all participants completed an informed consent form and preexercise health status questionnaire before participating. The purpose of these forms were to ensure that the rights of the participants were protected, to screen out participants that may be at risk for injury, and to determine the training age of the participants.

## Subjects

Twenty healthy males, from the University of Oklahoma, Norman Campus, participated in this investigation and were split into two groups (novice and advanced) based on their preexercise health status questionnaire answers. Specifically, participants that had aerobically trained ≤ 2 days/week, for ≤ 3 months, were placed in the novice training age group; while subjects that aerobically trained ≥ 4 days/week, for ≥ 1 year, were placed in the advanced training age group. Furthermore, all participants were recreationally active for approximately an hour each day of exercise.

### Aerobic exercise intervention

Seven visits (one initial screening visit, two *V*O_2_ peak test visits, and four RPP visits) were required for the completion of this investigation. Specifically, during the initial screening visit, but after the initial screening process, the investigator recorded the participant's resting measurements (see Table 1), and seat height on the upright cycle ergometer (model 906900, Lode B. V. Medical Technology, Groningen, the Netherlands). Next, the investigator had the participants cycle at 50 W for 10 min, using only their DOM limb (determined by which limb participants choose to kick a ball), while the N‐DOM limb rested on an adjacent, square box (purpose was to ensure the box height allowed the participant's limbs to align correctly during cycling) so as to not to contribute to the cycling action of the DOM limb. In addition, the participant's position on the bike was one in which the participant's DOM limb was securely fastened to the pedal, and almost fully extended at its lowest position. Furthermore, there were no counter‐balancing weights on the N‐DOM limb's pedal (because pedal was removed to allow for the placement of the adjacent, square box for the N‐DOM limb) to aid the DOM limb during exercise, all exercise visits were separated by 48 h, all participants refrained from consuming, or using, any item that could be considered as an ergogenic aid, all participants abstained from exercise for a minimum of 4 h prior to each visit (due to visits beginning in the morning), and all visits had room conditions between 20° and 23°C (68°–74°F).

**Table 1:** Subject group characteristics

Variables	Novice trained	Advanced trained
Age (yr)	22.9 ± 3.5	22.8 ± 2.6
Height (cm)	181 ± 7.5	180 ± 5.9
Weight (kg)	87.2 ± 10.7	85.2 ± 10.5
BMI (kg/m^2^)	26.6 ± 3	26.2 ± 2.2
Dom Thigh Skinfold (mm)	15.1 ± 4.6	14.55 ± 5.3
Non‐Dom Thigh Skinfold (mm)	15.55 ± 3.5	15.2 ± 3.8
*V*O_2_ peak (ml/kg/min)	25.1 ± 4.3	44.7 ± 3.7

yr = year; cm = centimeters; kg = kilograms; m = meter; mm = millimeter; mL = milliliter; min = minutes.

During the 1st and 2nd visits, the participants performed two separate, one‐leg *V*O_2_ peak tests, while pulmonary gas exchange was measured, using a metabolic cart (TrueOne^®^ 2400 model, Parvo Medics, Sandy, UT). The gas analysis system was calibrated (prior to testing) following the instructions from the manufacturer. Furthermore, the one‐leg *V*O_2_ peak testing protocol was the same protocol used by McPhee et al. (2009). Specifically, the warm‐up phase lasted two min, while the participants cycled at 20 W. Following this warm‐up phase, the external workload increased to 40 W for one min, and kept increasing by 10 W, every subsequent min, until the participants could no longer maintain a cadence of 70 rev/min, or stopped due to volitional exhaustion. After the one‐leg *V*O_2_ peak test was complete, participants had a cool‐down phase that lasted 2 min at 40 W. Most importantly, one‐leg *V*O_2_ peak values were recorded from the highest 30 sec average oxygen uptake during the 2nd visit.

**Figure 1:**
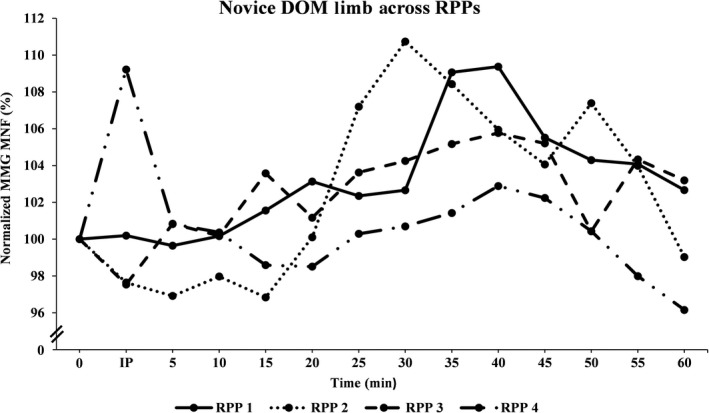
Represents the pooling of all mechanomyographic (MMG) MNF values from the DOM VLM, for the novice group across time. 0 =  Baseline (pre‐exercise) measurement; IP = Immediately postexercise measurement; resting postural positions (RPP) 1 =  Upright sitting position with legs extended 180°; RPP 2 =  Upright sitting positon with legs bent 90°; RPP 3 =  Lying supine position with legs extended 180°; RPP 4 =  Lying supine position with legs bent 90°.

**Figure 2:**
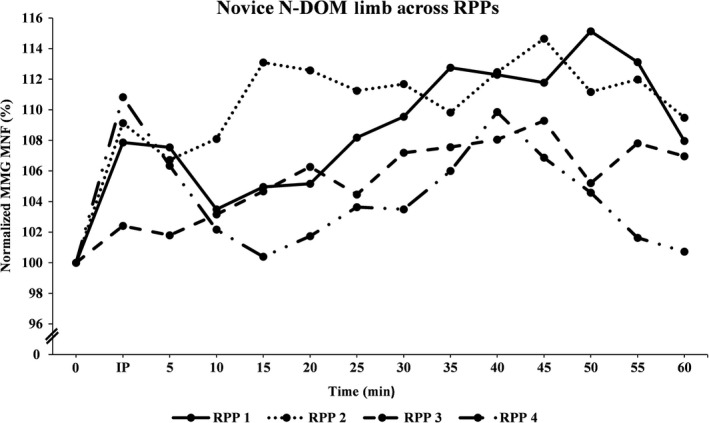
Represents the pooling of all mechanomyographic (MMG) MNF values from the N‐DOM VLM, for the novice group across time. 0 =  Baseline (preexercise) measurement; IP = Immediately postexercise measurement; resting postural positions (RPP) 1 =  Upright sitting position with legs extended 180°; RPP 2 =  Upright sitting positon with legs bent 90°; RPP 3 =  Lying supine position with legs extended 180°; RPP 4 =  Lying supine position with legs bent 90°.

**Figure 3:**
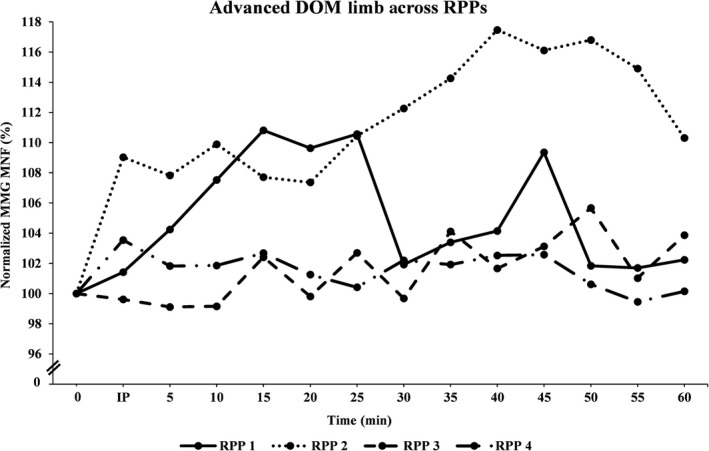
Represents the pooling of all mechanomyographic (MMG) MNF values from the DOM VLM, for the advanced group across time. 0 =  Baseline (preexercise) measurement; IP = Immediately postexercise measurement; resting postural positions (RPP) 1 =  Upright sitting position with legs extended 180°; RPP 2 =  Upright sitting positon with legs bent 90°; RPP 3 =  Lying supine position with legs extended 180°; RPP 4 =  Lying supine position with legs bent 90°.

**Figure 4:**
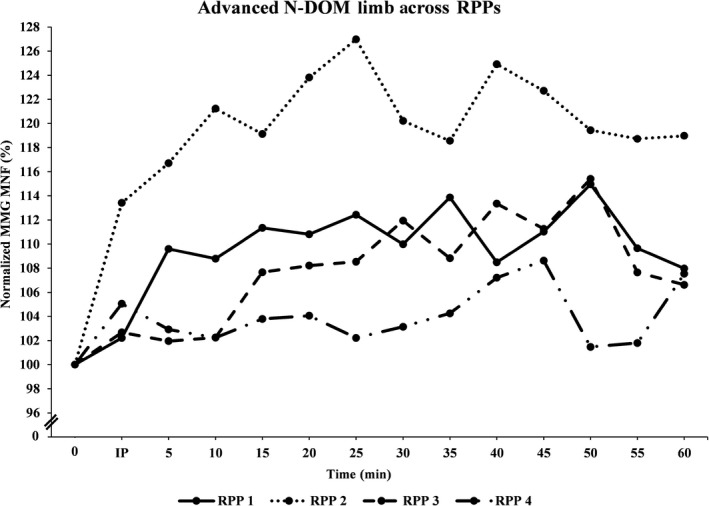
Represents the pooling of all mechanomyographic (MMG) MNF values from the N‐DOM VLM, for the advanced group across time. 0 =  Baseline (preexercise) measurement; IP = Immediately postexercise measurement; resting postural positions (RPP) 1 =  Upright sitting position with legs extended 180°; RPP 2 =  Upright sitting positon with legs bent 90°; RPP 3 =  Lying supine position with legs extended 180°; RPP 4 =  Lying supine position with legs bent 90°.

During the last four visits, the participants either sat/laid, on a padded chair/table, in one‐of‐four RPPs for sensor attachment over both vastus lateralis muscles (VLMs). Specifically, the RPPs were randomly assigned for each visit and included: (1) sitting upright, while both limbs were extended 180°; (2) sitting upright, while both limbs were bent at 90°; (3) lying supine, while both limbs were extended 180°; or (4) lying supine, while both limbs were bent at 90°. The purpose of these four different RPPs was to modify the muscular length of the VLM. Furthermore, for the RPPs that required a knee flexion of 90°, the participants were asked to firmly place their feet onto a square box (same as the one used during unilateral cycling) to ensure that their limbs maintained a 90° flexion at the knee. MMG and sEMG sensors were attached over both VLMs (purpose of sEMG sensors were to ensure that both VLMs remained relaxed during all measurements), and a permanent marker was used to mark the locations on the skin (purpose was to ensure repeated accuracy of sensor placement across all visits). After skin preparations were complete, participants sat/laid quietly for approximately 5 min in one‐of‐four RPPs, and measurements were taken for one min to establish a resting baseline for the MMG MNF data. Following those measurements, the sEMG sensor from the N‐DOM limb remained connected, while all other sensors were disconnected (purpose of sEMG sensor was to ensure that the N‐DOM limb remained relaxed during cycling). Next, participants began cycling for 2 min at a power wattage, using 30% of their *V*O_2_ peak. After the warm‐up phase was complete, participants cycled for 30 min at 60% of their *V*O_2_ peak. Following this aerobic phase, participants cooled down for two min at 30% of their *V*O_2_ peak. Once the cool‐down phase was complete, participants returned to the same RPP as with their baseline measurements. The DOM limb's sEMG sensor and both limb's MMG sensors were reattached, and signals were recorded from both VLMs every 30 sec, for each min, for the next 60 min. Hence, each participant's sEMG and MMG signals were digitally saved in a lab computer every 30 sec, for each min, for the subsequent 60 min. However, our results will be explained in relation to RPP (and in a few instances with 5 min interval values [i.e., Figures 1‐4]), due to the overwhelmingly large number of recorded signals from each VLM. Most importantly, the primary investigator encouraged all participants to remain as motionless as possible during all pre and postexercise measurements, across all visits. Additionally, the primary investigator attentively monitored all data to ensure that it was free from any movement artifact (i.e., breathing heavy, twitching, shifting, etc.), and once all measurements had been recorded, all sensors were disconnected and the participants were dismissed from the lab.

### Electromyography and mechanomyography measurements

Prior to placement of both sensors and the reference electrode, skin over both VLMs and the 7th cervical vertebrae were prepared by careful shaving and cleansing with alcohol. Specifically, the sEMG (DE 2.1 single differential surface EMG sensor of 10 mm interelectrode distance; Delsys, Inc., Boston, MA) and MMG (Entran EGAS FT‐10; Measurement Specialties, Hampton, VA) sensors were placed over both VLMs, while the reference electrode was placed over the C7 vertebrae according to the procedures described in the SENIAM project (Hermens et al. 1999). More specifically, the sensors for the VLM were placed on a line, two‐thirds of the way from the anterior spina iliaca, superior to the lateral side of the patella, in the direction of the muscle.

### Signal processing

Analog sEMG signals (baseline, exercise, and postexercise) were preamplified (gain = 1000) with a modified Bagnoli 16‐channel EMG system (Delsys, Inc.), digitized at a rate of 20,000 samples/sec, by a 12‐bit analog‐to‐digital converter (National Instruments, Austin, TX), and stored in a personal computer (Dell Optiplex 755, Round Rock, TX) for subsequent analyses. The sEMG signals were then digitally band‐pass filtered (4th–order Butterworth) with pass frequencies, between 10 and 500 Hz.

Analog MMG signals (baseline and postexercise) were digitized at a rate of 1000 samples/sec, by a 12‐bit analog‐to‐digital converter (National Instruments, Austin, TX), and stored in a personal computer (Dell Inspiron, Latitude D620, Round Rock, TX) for subsequent analyses. The MMG signals were then digitally band‐pass filtered (4th–order Butterworth) with pass frequencies, between 5 and 50 Hz.

The Discrete Fourier Transform (DFT) algorithm was used to derive the sEMG and MMG power spectrum (purpose was to calculate the MNF based on the equation described by Kwatny et al. 1970). Furthermore, all sEMG and MMG MNF values, for each muscle, were then normalized as a percentage of their respective baseline (preexercise) measurements. Lastly, sEMG and MMG signal processing was performed with two separate, custom programs written with LabVIEW programming software (version 7.1, National Instruments, Austin, TX).

### Data analysis

One, four‐way (training group x muscle x resting postural position x time) repeated measures, analysis of variance (ANOVA) was performed to analyze the MMG MNF data. When appropriate, follow‐up analyses included: three‐way, two‐way, and one‐way repeated measures ANOVAs, paired samples t‐tests, and bivariate correlations with Bonferroni post hoc comparisons. In addition, effect sizes (ES) were determined using Cohen's *d* and eta squared. Specifically, for determining Cohen's *d*, we used the following equation,

$$d=\left(Y_{1}-Y_{2}\right)/S_{p}$$

and the proposed standards for interpretation of *d* (small ES = 0.2; moderate ES = 0.5; large ES = 0.8). Furthermore, for determining eta squared, we used the following equation,

$$\eta^{2}=t^{2}/\left(t^{2}+\text{df}\right)$$

and the proposed standards for the interpretation of ɳ^2^ (small ES = 0.01; moderate ES = 0.06; large ES = 0.14). An a priori sample size estimation, using G*Power 3.1 software, indicated that for an alpha level of 0.05 and a power level of 0.80, a sample size of approximately 20 participants (10 in each group) was appropriate. All statistical analyses were performed, using the Statistical Package for the Social Sciences (SPSS) version 22 for Windows, with a critical alpha of *P* < 0.050.

## Results

All resting measurements (baseline, exercise, and postexercise) of sEMG voltage were less than one microvolt, which was below the preestablished system noise cutoff of 1.2 microvolts. Thus, any sEMG measurements below that cutoff point could not be considered statistically different from random. Therefore, due to the recording of those low voltage measurements, we did not further analyze the sEMG signals.

**Figure 5:**
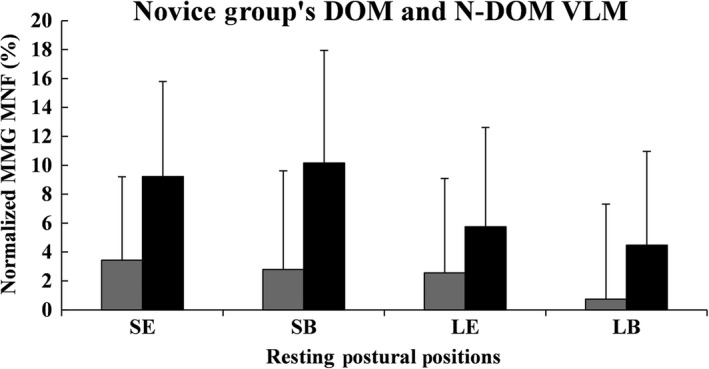
Represents the pooling of all mechanomyographic (MMG) MNF values from the DOM and N‐DOM VLMs, for the novice group. The DOM VLM is depicted by gray rectangles, while the N‐DOM VLM is depicted by black rectangles. SE = Upright sitting position with legs extended 180° (RPP 1); SB = Upright sitting positon with legs bent 90° (RPP 2); LE = Lying supine position with legs extended 180° (RPP 3); LB = Lying supine position with legs bent 90° (RPP 4).

**Figure 6:**
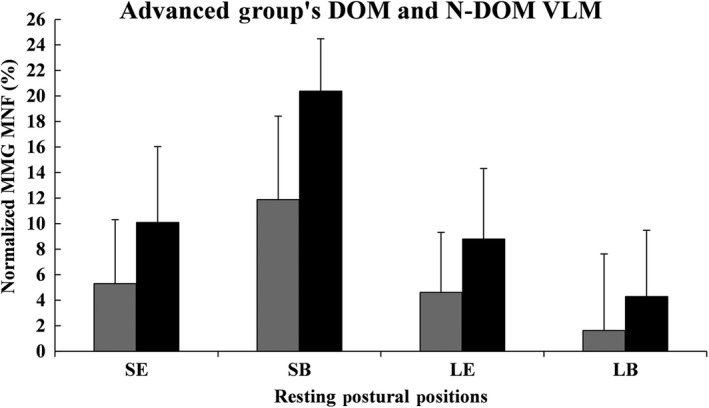
Represents the pooling of all mechanomyographic (MMG) MNF values from the DOM and N‐DOM VLMs, for the advanced group. The DOM VLM is depicted by gray rectangles, while the N‐DOM VLM is depicted by black rectangles. SE = Upright sitting position with legs extended 180° (RPP 1); SB = Upright sitting positon with legs bent 90° (RPP 2); LE = Lying supine position with legs extended 180° (RPP 3); LB = Lying supine position with legs bent 90° (RPP 4).

Figures 1, 2, 3, 4, 5, 6 show the mean (±SD) normalized MMG MNF values, for both groups, from their respective DOM and N‐DOM VLM, at each RPP. Specifically, for the novice group there were approximate increases of 3.4 ± 5.8% and 9.2 ± 6.6%; 2.8 ± 6.8% and 10.2 ± 7.8%; 2.6 ± 6.5% and 5.8 ± 6.9%; and 0.7 ± 6.6% and 4.5 ± 6.5% (for RPPs 1–4, respectively). For the advanced group, there were approximate increases of 5.3 ± 5% and 10.1 ± 5.9%; 11.9 ± 6.5% and 20.4 ± 4.1%; 4.6 ± 4.7% and 8.8 ± 5.5%; and 1.6 ± 6% and 4.3 ± 5.2% (for RPPs 1–4, respectively), from the DOM and N‐DOM VLM, respectively. Furthermore, the difference between the training group's DOM limbs were approximately 1.9 ± 0.8%, 9.1 ± 0.3%, 2 ± 1.8%, and 0.9 ± 0.6% (for RPPs 1–4, respectively), while for the N‐DOM limbs there were approximate differences of 0.9 ± 0.7%, 10.2 ± 3.7%, 3 ± 1.4%, and 0.2 ± 1.3% (for RPPs 1–4, respectively). Thus, Figures 1, 2, 3, 4, 5, 6 represent the pooling of all data points from each VLM across the four RPPs, respectively.

### ANOVA analyses

The results from the four‐way repeated measures ANOVA for MMG MNF indicated a statistically significant (*P* < 0.050) main effect for muscle, resting postural position, and time (ɳ^2^ = 0.305; ɳ^2^ = 0.303; and ɳ^2^ = 0.360, respectively). As for the main effect for muscle, since we only had two muscles to compare (DOM vs. N‐DOM), a paired samples t‐test was considered appropriate to be performed, and the results indicated that there was a statistically significant (*P* < 0.050) mean difference between VLMs (*d *=* *0.53). Furthermore, for the main effect for resting postural position, a one‐way repeated measures ANOVA with Bonferroni post hoc comparisons was performed, and the results indicated that there were statistically significant (*P* < 0.050) mean differences between resting postural positions (ɳ^2^ = 0.885). An additional paired samples t‐test was performed, and the results indicated that there were statistically significant (*P* < 0.050) mean differences between RPPs 1 and 2 (*d *=* *1.24); 1 and 3 (*d *=* *4.05); 1 and 4 (*d *=* *3.37); 2 and 3 (*d *=* *2.62); and 2 and 4 (*d *=* *2.23). Moreover, for the main effect for time, a one‐way repeated measures ANOVA with Bonferroni post hoc comparisons was performed, and the results indicated that there were statistically significant (*P* < 0.050) mean differences across time (ɳ^2^ = 0.262). Another follow‐up paired samples t‐tests (see Table 2) indicated that there were statistically significant (*P* < 0.050) mean differences between pre and postmeasurements, as well as between sequential postexercise measurements.

**Table 2:** Main effect for time

	***P*** **‐value**	**Cohen's** ***d***
Pre versus postexercise time points (min)		
15	0.026	1.19
20	0.020	1.26
25	0.016	1.32
30	0.011	1.42
35	0.005	1.68
40	0.003	1.78
45	0.004	1.73
50	0.005	1.63
55	0.020	1.26
60	0.049	1.02
IP versus postexercise time points (min)		
40	0.045	0.92
50	0.049	0.81
Post 5 versus postexercise time points (min)		
35	0.033	0.70
40	0.028	0.86
50	0.027	0.75
Post 15 versus postexercise time points (min)		
35	0.036	0.53
40	0.036	0.71
Post 20 versus postexercise time points (min)		
30	0.012	0.29
35	0.011	0.46
40	0.016	0.64
Post 30 versus postexercise time points (min)		
60	0.019	0.38
Post 35 versus postexercise time points (min)		
60	0.014	0.55
Post 40 versus postexercise time points (min)		
45	0.031	0.22
55	0.029	0.43
60	0.011	0.72
Post 50 versus postexercise time points (min)		
55	0.029	0.30
60	0.033	0.60

Pre = Baseline measurement; IP = Immediate postexercise measurement.

Lastly, a bivariate correlation analysis was performed between both VLMs at each RPP, for both groups. Specifically, for the novice group there was a statistically significant (*P* < 0.050) correlation between both VLMs during RPPs 2 and 4 (*d *=* *0.41 and *d *=* *0.15, respectively); with the correlation being considered moderate for both RPPs (*r* = 0.67 and *r* = 0.65, respectively). In addition, for the advanced group there was also a statistically significant (*P* < 0.050) correlation between both VLMs during RPPs 2 and 4 (*d *=* *0.43 and *d *=* *0.21, respectively); however, these correlations were considered moderate for RPP 2 (*r* = 0.58), and strong for RPP 4 (*r* = 0.91).

## Discussion

Our present results demonstrated that there was a statistically significant (*P* < 0.050) increase in normalized MMG MNF postexercise. Specifically, we observed an increase in the resting normalized MMG MNF values for both VLMs from both groups, despite an absence in resting sEMG signals. More specifically, when assessing the increases in normalized MMG MNF between both groups, we observed that with the novice group, the large increase in the N‐DOM VLMs MMG MNF, for all RPPs (except RPP 3), was accompanied by a granular increase in the DOM VLMs MMG MNF. Interestingly, the advanced group had greater normalized MMG MNF values (when compared to the novice group) from both VLMs during all RPPs (except RPP 4). Although, we did notice that with this large increase in the N‐DOM VLM MMG MNF, there was a moderate increase in the DOM VLMs MMG MNF. Nevertheless, we did find a similar response between training groups, in that, we observed an increase in ipsilateral and contralateral MMG MNF, with the N‐DOM VLM having the larger increase in MMG MNF. However, due to each groups individual results, we believe that despite the relative matching of exercise intensity between groups, acute responses to contralateral training still appear to be dependent upon training age/fitness level (i.e., cross‐over effects becomes less accentuated as one progresses in training age/fitness level).

Until recently, conventional researchers believed that for any muscle to be active, it must receive electrical activation (through the motor neuron pool, or bypassed via external stimulation) and should be able to be clearly recorded from any good quality sEMG system. However, previous researchers (McKay et al. 2004, 2007; Wages et al. 2015, 2016a) have found that a lack of sEMG voltages in a resting muscle is not a determinant for a lack of muscular activity. Specifically, our present data is in alignment with our previous research, as well as their previous findings, in that, we observed sEMG voltages that were less than one microvolt. Therefore, based on the McKay group's original recommendations and conclusions (McKay et al. 2004), we could not consider our subject's sEMG values statistically different from random. Due to this statistical insignificance, we did not perform any additional follow‐up sEMG analyses.

As similarly reported in our previous investigation (Wages et al. 2015), we need to also recognize that the MMG signals from the VLM may be partially reflective of the MMG activity from an adjacent, synergist muscle (potentially the rectus femoris muscle [RFM] since it is a biarticular [crossing two joints] muscle). However, even though we believe that this potential “cross‐talk” between muscles is highly doubtful due to the relative size difference between both muscles (i.e., RFM is approximately half the volume of the VLM), we cannot disregard the possibility that the MMG sensor over the VLMs may have inadvertently “picked‐up” an inconsequential amount of additional resting MMG signals. This may have potentially occurred due to the relative distance between both muscles, their respective proximal and distal attachments, as well as their muscular lengths associated with each RPP. Nevertheless, if there was a small degree of “cross‐talk” between muscles, it would not greatly impact, or influence, the signals detected from the VLMs (i.e., we would still observe a statistically significant increase in MMG MNF).

Now, when taking into account the results from our present investigation, we must confer with the hypothesis first presented by the authors of the Carroll et al. (2006) article, in that, there must be multiple, overlapping neural (in conjunction with mechanical) mechanisms being concurrently activated, across the training and recovery periods. However, as speculated by those previous authors, the ability to potentially differentiate between the specified temporal inputs from either mechanism is relatively challenging without the proper equipment (i.e., functional MRI, ultrasound, NIRS, etc.). Nevertheless, previous researchers (Zhou 2000; McKay et al. 2004, 2006, 2007; Carroll et al. 2006; Beck 2010) have listed a series of possible independent mechanisms (mechanical and neural) that may be coactively responsible for contralateral responses or adaptations within resting musculature, postexercise. More specifically, associated mechanical influences may be a result of changes to muscular temperature, muscular stiffness, muscle/adipose mass, intramuscular pressure, viscosity of the intracellular and extracellular fluid mediums, muscle pump from blood pooling, or velocity of blood flow; while associated neural influences may be due to the diffusion of impulses between cerebral hemispheres, coactivation via bilateral corticospinal pathways, postural stabilization, altered sensitivity of muscle spindles or Golgi tendon organs, facilitation of gamma loop reflexes at the cerebral, spinal or peripheral level, or altered actin‐myosin cross‐bridge formation. Additionally, these collaborative efforts amongst mechanical and neural mechanisms may also be subject to potentially change dependent upon the age of the participant, the time course of relaxation, muscle fiber composition, muscle/tendon length ratio, or if the resting muscle is in a lengthened or shortened state.

Therefore, we have reasoned that our present results could be potentially attributed to the notion that immediately following the conclusion of unilateral fatiguing exercise, and independent of musculature length, muscle temperature is still elevated, the exercised muscle is severely fatigued, and the intramuscular pressure is still high due to the intermittent bouts of blood flow restriction sustained during the repetitive actions of cycling. Within a few minutes, the intramuscular pressure is greatly reduced and regular blood flow is restored, but at an increased velocity. Since the muscle is no longer exercising, this increased velocity will eventually cause the pooling of blood in the lower extremities, thus prompting the fibers around the veins to intermittently constrict for the purpose of helping shuttle the blood back toward the heart (Beck 2010). Now, if we were to modify the hip and/or knee joint angle, there may be a change in the length of the resting muscle, which may cause a high degree of muscular stiffness in association with the above mechanical conditions. Furthermore, at approximately the same time there is most likely an extended hyperexcitability for the diffusion of impulses between the cerebral hemispheres (due to the activation of the motor cortex in one hemisphere being active during unilateral fatiguing exercise), as well as the an increased coactivation of a bilateral corticospinal pathways (due to a large percentage [~90%] of impulses from the motor cortex being conducted to the spinal cord through the lateral and anterior corticospinal tract of the contralateral side, and a small percentage [~10%] remaining on the ipsilateral side [Carroll et al. 2006]). Thus, if we were to combine all of the above mechanical and neural conditions, we would potentially observe an increase in the MMG MNF values for not only the resting ipsilateral muscle, but also the resting contralateral muscle across a postexercise time period of recovery.

Another possible explanation for our ipsilateral results could be potentially due to how the recruitment/decruitment and firing rates behaviors of motor units modify their activity from when a muscle is less fatigued to when it is greatly fatigued. As stated by previous authors (Adam and De Luca 2005; Contessa and De Luca 2013; Contessa et al. 2016), the central nervous system changes the operating point of the excitation for an active motoneuron pool to compensate for, and simultaneously remain highly sensitive to, the changes in firing rate behavior and muscle force twitch. Thus, the excitation to the active motoneuron pool decreases from the onset to the conclusion of fatiguing exercise. As a result, previously active motor units are decruited earlier, while at the same time new motor units are recruited earlier to help maintain a constant force output during fatiguing exercise. Therefore, the overall number of active motor units may stay approximately the same, but the firing rates from the active motor units may be variable due to the recruitment of higher threshold motor units toward the latter half of the fatiguing exercise intervention. Since motor unit behavior is based on a continuum‐type paradigm, the operating point of sensitivity for the motoneuron pool must shift toward the right to accommodate for those newly recruited higher threshold motor units. This means that when the fatiguing bout of exercise is complete, the operating point must shift back to the left, toward the homeostatic sensitivity of when the muscle was “fresh” (not fatigued). As a result, the AMP component of the sEMG signal is severely reduced (possibly due to the decruitment of higher threshold toward lower threshold motor units), which may cause the associated AMP component of the MMG signal to also be acutely reduced across the recovery period (which could potentially cause the MMG MNF to subsequently increase). And again, the ensuing contralateral results would be potentially due to the extended hyper‐excitability for the diffusion of impulses between the cerebral hemispheres, as well as the increased coactivation of a bilateral corticospinal pathways.

### Possible weakness of the investigation

We did not measure changes in muscular strength (i.e., pre vs. immediately postexercise) for either limb. By assessing the exact degree of strength loss for both limbs, we would have been able to completely ensure that a clear “mechanical” effect was present across the exercise and recovery time periods. As a result, we would have also been able to potentially provide evidence for a direct correlation between MMG responses and a certain value of strength loss. However, if we were to perform a strength test following fatiguing aerobic exercise, that particular act (transitioning from aerobic to resistance exercise) would have most likely negated any of our present results due to the associated change of task performance, as well as the input modifications from the central and peripheral nervous systems.

Furthermore, we also did not measure changes in muscle temperature between limbs across the exercise, or recovery time course. By assessing the exact changes in temperature for both limbs, we would have been able to explore the possible role of resting muscle mechanical activity in maintaining the thermoregulatory tonus of a muscle. As a result, we would have also been able to potentially provide evidence as to how changes in muscular lengths affect the cooling rate of muscle, as well as being able to provide a possible explanation for how temperature changes of the DOM, EXL affects the MMG response of the N‐DOM, N‐EXL.

## Conclusion

Our results rejected our main hypothesis (e.g., the novice group having the higher MMG MNF values in both limbs, across all four RPPs). However, even though the advanced training age group had higher normalized MMG MNF values for both limbs (for nearly all RPPs), their relative percent change difference between limbs was lower than that found with the novice training age group. Hence, this finding supports the “traditional theory” or “conventional wisdom” regarding contralateral cross‐over effects being greater for a novice trained group (when compared to an advanced trained group) postexercise. Furthermore, our results provide contributing support to “contemporary knowledge” that a lack of sEMG signals in a resting muscle is not a determinant for a lack of muscular activity. In addition, our results also lend subsequent evidence to the notion (as first presented by Carroll et al. 2006) that the human body concurrently activates the utilization of multiple (and most likely interchangeable) mechanical and neural mechanisms over an extended recovery time course following exercise (instead of enlisting their efforts separately, as previously thought).

It is also important to note that our results are the first to provide evidence of contralateral cross‐over effects occurring in an advanced training aged population across an acute recovery time period. Furthermore, we are the first to suggest a new potential explanation (based on recent sEMG literature [Adam and De Luca 2005; Contessa and De Luca 2013; Contessa et al. 2016]), as to why these acute ipsilateral MMG MNF responses may have occurred (i.e., the hypothesis related to the transitional shifting of the body's operating point to maintain a constant force during fatiguing exercise). Additionally, our results have suggested that despite the relative matching for the “stressor or stimulus” needed to provoke similar responses to acute exercise (based on relative exercise intensity for each respective training group), training age/fitness level appears to be the main determining factor for the overall changes related to acute ipsilateral and contralateral responses. Lastly, as new physiological techniques become more commercialized in the near future, it will be possible to subject these different mechanical and neural mechanisms to greater scrutiny to either confirm or refute their degree of potential influence across the recovery time frame following fatiguing exercise.

## Submission Declaration

We represent that this submission is the original work, not previously published and is not under consideration for publication with any other journal.

## Conflict of Interest

We represent that there are no actual or potential conflicts of interest including any financial, personal, or other relationships with people or organizations.
